# Dairy and Plant-Based Dairy Alternative Consumption Across Food-Related Consumer Segments: Food Involvement, Sustainability Orientation, and Health-Oriented Profiling

**DOI:** 10.3390/nu18132135

**Published:** 2026-07-02

**Authors:** Sylwia Żakowska-Biemans

**Affiliations:** Department of Food Market and Consumer Research, Institute of Human Nutrition Sciences, Warsaw University of Life Sciences—SGGW, Nowoursynowska 159c, 02-776 Warsaw, Poland; sylwia_zakowska_biemans@sggw.edu.pl

**Keywords:** consumer segmentation, plant-based dairy alternatives, food involvement, sustainable food choices, dairy consumption, lifestyle, meat reduction

## Abstract

**Background/Objectives:** Consumption of dairy products and plant-based dairy alternatives (PBDAs) can be examined within broader configurations of food-related orientations rather than as isolated product choices. This study aimed to identify food-related consumer segments based on food involvement, attention to on-pack product information, and sustainability-related food-choice orientations, and to characterise these segments in relation to reported consumption frequencies of dairy products, PBDAs, and meat, fish and legume dishes, as well as health-oriented food-choice criteria. **Methods:** A cross-sectional survey was conducted among 1508 Polish adults responsible or co-responsible for household food purchasing. Principal component analysis was used to identify underlying food-related dimensions, and the retained component scores were entered into a two-step cluster analysis. Differences between clusters were examined using chi-square tests and one-way ANOVA. **Results:** Six dimensions were retained: sustainable and ethical choices, meat reduction, food involvement, product-information importance, shopping-list use and food-waste avoidance. Five clusters were identified, reflecting distinct configurations of these dimensions. PBDA and legume-dish consumption were most frequent in the sustainability and meat-reduction-oriented cluster, although dairy products and meat remained part of the reported diet. High food involvement and label/quality attention co-occurred with a more conventional consumption pattern, whereas PBDA and legume-dish consumption were lowest in more conventional and lower-sustainability clusters. The low-engagement cluster showed a more selective pattern of PBDA and legume-dish consumption. **Conclusions:** This study identified five food-related consumer segments and showed that reported PBDA consumption was embedded in heterogeneous dietary patterns rather than functioning as a simple substitute for dairy products. These findings indicate that reported PBDA consumption is segment-dependent and cannot be assumed to reflect reduced dairy consumption or a consistently sustainability- or health-oriented dietary pattern.

## 1. Introduction

Consumer choices involving dairy products are increasingly discussed in relation to health, ethical and sustainability-related concerns [1,2,3,4]. Dairy products remain deeply embedded in everyday dietary patterns, whereas plant-based dairy alternatives (PBDAs) have become an increasingly accessible and diverse product category [5,6,7,8]. At the same time, the relationship between dairy consumption and plant-based alternatives should not be reduced to a simple substitution pattern. Evidence from different studies shows coexistence, complementarity, dual-use patterns, and distinct motivations, rather than pure replacement [9,10]. Consumers may integrate dairy products and plant-based alternatives into their diets in different ways, depending on product type, consumption occasion, perceived naturalness, nutritional value, taste expectations, and trust in product information [11,12,13]. Previous studies have shown that consumers differ not only in their liking of dairy milk and plant-based alternatives, but also in their sensory, emotional, cognitive, and situational responses to these products [6,14,15]. Food involvement offers a relevant framework for explaining this heterogeneity. Food involvement refers to the degree to which food is personally relevant and important in everyday life, including affective engagement with food, enjoyment of eating and drinking, the perceived importance of food-related decisions, and the role of food in social life [16,17,18]. Consumers with higher levels of food involvement are therefore more likely to attend to product information, compare labels and assess products using multiple criteria, including health, naturalness, environmental impact and ethical attributes [16,18].

In the dairy and PBDAs category, such evaluations are particularly complex because consumers are exposed to competing narratives about nutritional value, naturalness, sustainability, animal welfare and the degree of processing [8,19,20,21,22]. Health concerns are particularly salient in this category, as both dairy products and their plant-based counterparts are commonly evaluated in relation to nutritional quality, perceived health benefits, and product composition. Although PBDAs are often positioned as alternatives to dairy products, their suitability as substitutes requires careful evaluation in terms of product formulation, sensory quality, environmental impact, labelling practices, and nutritional comparability. Plant-based milk alternatives may differ from dairy milk in protein content and in the levels of micronutrients such as vitamin B2, vitamin B12, calcium, and iodine, while some plant-based cheese alternatives may contain relatively high levels of saturated fat or sodium [23,24,25]. Health-oriented choice criteria, therefore, provide a relevant basis for understanding consumer differentiation in this product category [3]. Alongside health-related considerations, sustainability constitutes another important dimension through which consumers evaluate dairy products and PBDAs. Environmental claims are frequently used to position PBDAs in relation to dairy products [26], but sustainability perceptions in this category are multidimensional and may involve different, and sometimes conflicting, consumer interpretations [27]. Sustainability-related concerns may influence how consumers interpret product attributes, compare dairy and non-dairy options, and justify their food choices [8,28]. They are therefore relevant not only as general attitudes, but also as factors that may differentiate consumer groups in terms of their perceptions, preferences, and behaviours toward dairy products and their plant-based counterparts. Taken together, these considerations indicate that consumer responses to dairy products and plant-based alternatives should be understood in relation to heterogeneity in food-related engagement and sustainability-related priorities. Segmentation provides a useful framework for developing a more differentiated understanding of consumer responses to dairy products and plant-based alternatives. It allows the analysis to move beyond product choice alone and to examine how patterns of dairy and PBDA consumption are embedded in broader food-related orientations, sustainability-related priorities, and dietary preferences. The present study contributes to this literature by examining consumer segmentation in the context of dairy products and PBDAs. Unlike approaches focused solely on the adoption of plant-based alternatives, this study integrates food involvement, label use, health-oriented choice criteria, sustainability-oriented choice criteria, and consumption frequency of selected food products.

The aim of this study was to (1) identify and describe consumer segments based on food involvement, attention to on-pack product information, and sustainability-related food-choice orientations, and (2) subsequently profile these segments in terms of reported consumption of dairy products, PBDAs, meat, fish and legumes, as well as health-oriented food-choice criteria. By linking orientation-based segmentation with consumption-based profiling, the study examines whether PBDA consumption co-occurs with broader patterns of dietary diversification, meat-reduction orientation and sustainability-oriented food choices, and whether these patterns are further differentiated by health-related food-choice criteria.

## 2. Materials and Methods

### 2.1. Participants

The study was conducted on a nationwide sample of Polish adults selected to reflect the population distribution in terms of gender, age, education and size of place of residence. Quotas were established on the basis of data from the 2021 National Population and Housing Census. Eligibility was limited to individuals aged 18 years or older who were responsible or co-responsible for household food-purchasing decisions. Data were collected using the computer-assisted web interview (CAWI) technique. A total of 1508 participants were recruited from ePanel.pl, an online access panel owned and administered by ARC Rynek i Opinia, a professional market research company. At the time of the study, the panel included approximately 50,000 registered users. Panel members were recruited through both online and offline channels and did not originate from targeted customer databases. The panel provider applied respondent verification procedures and quality-control standards, and participants who met the recruitment criteria and completed the questionnaire received a monetary incentive depending on survey length. The study followed the ethical principles of the Declaration of Helsinki and received ethical approval from the Rector’s Committee for the Ethics of Research Involving Human Participants at Warsaw University of Life Sciences on 5 July 2024 (protocol no. 31/RKE/2024). Informed consent was obtained from all participants before data collection by the market research company administering the survey. Respondents were informed in advance about the nature of their participation and their rights, including the right to withdraw from the study at any stage. Before starting the questionnaire, participants were required to confirm that they understood the purpose of the study and agreed to participate by selecting a consent box. All data were encoded in a non-identifiable format and processed anonymously.

### 2.2. Measures

#### 2.2.1. Segmenting Variables

The segmentation was based on selected items adapted from established instruments measuring food-related lifestyle (FRL) [29,30], food involvement [18], and sustainable, healthy eating behaviours [31]. The item selection was theory-driven and purpose-specific. The aim was not to reproduce the full original scales, but to capture food-related orientations considered directly relevant to the study objective: understanding how dairy and plant-based dairy alternative consumption is embedded in broader patterns of food-related engagement, product information use, sustainability-related priorities, meat-reduction orientation, and household food management practices. Conceptually, the selected segmentation variables were expected to reflect six broad domains of food-related orientation: food involvement; product-information and label use; shopping planning; sustainable and ethical choice criteria; meat-reduction and plant-protein orientation; and food-waste avoidance. These dimensions were used to capture variation in food-related orientations that may contribute to a deeper understanding of dairy and PBDA consumption patterns, including attention to product information, perceived quality, sustainability, animal welfare, meat reduction, and the patterns of animal- and plant-based food consumption [27,28,32,33,34,35]. Food involvement captured the affective and personal relevance of food, while product information and label use reflected cognitive reliance on informational cues, including nutritional value, ingredients, production methods, certification, and sustainability-related attributes. Shopping planning represented the organisational dimension of household food management and may indirectly support sustainability-related practices by promoting more deliberate purchasing and food-waste prevention [36]. Meat-reduction and plant-protein orientation were included to position dairy and plant-based dairy alternative choices within broader animal- and plant-based food choice patterns [13,37,38]. Food-waste avoidance was included as a responsibility- and sustainability-oriented dimension of household food practices [39].

Items related to label use and product information were adapted from the FRL framework, mainly from the “Ways of Shopping” domain and the “importance of product information” dimension [29]. These items assessed the extent to which respondents use food labels, package information and nutritional information when making food choices. Additional items concerning the use of shopping lists were also derived from the FRL “Ways of Shopping” domain, where shopping planning is treated as part of habitual food-purchasing practices. Food involvement was measured using items from the Core Food-Related Lifestyle instrument [18]. They referred to the enjoyment of good food, the role of food and drink as a source of pleasure, the perceived importance of decisions about eating and drinking, and the role of food in everyday and social life. Items reflecting sustainability-oriented food choice criteria were selected from the Sustainable Healthy Eating scale [31]. These criteria included attention to quality and regional certifications, organic and regional food, environmentally friendly production, the use of legumes and plant-based foods as alternatives to meat, food waste avoidance, and animal welfare considerations. All items were translated and adapted to the Polish survey context. Responses were measured on Likert-type or frequency scales, depending on the wording of the item and the construct being measured. The selected items were then subjected to principal component analysis to identify the underlying structure of food-related attitudes and choice criteria in the study sample. Component scores obtained from this analysis were subsequently used as input variables in the cluster analysis.

#### 2.2.2. Characterising Variables

After the clusters were identified, they were profiled using socio-demographic characteristics, health-oriented choice criteria, and frequency of food consumption variables. Food-consumption frequency was measured using selected items from the KomPAN questionnaire [40]. The selected food-consumption frequency items were chosen to cover key categories of dairy products and their principal PBDAs counterparts. Dairy categories included milk, fermented milk drinks, cottage/quark cheese, and Gouda-type cheese, while PBDA categories included plant-based milk alternatives, plant-based yoghurt/kefir alternatives, and plant-based cheese alternatives. These categories were selected because they represent commonly available and consumer-facing dairy and PBDAs product groups and allow for comparison between dairy products and their plant-based counterparts. The inclusion of meat, fish, and legumes was intended to contextualise dairy and PBDA consumption within broader patterns of animal- and plant-based food choice. Response categories ranged from “never” to “several times per day”. The health-oriented profiling items were selected from the Sustainable Healthy Eating scale [31]. They referred to choosing nutritious foods, foods that support health, foods rich in vitamins and minerals, foods without additives, avoiding sugar-sweetened beverages and limiting salt intake. Socio-demographic profiling variables included gender, age, education, place of residence, household size, presence of children under 18 in the household, and self-assessed financial situation.

### 2.3. Data Analysis

Data were analysed using descriptive statistics, principal component analysis (PCA), two-step cluster analysis, one-way analysis of variance (ANOVA), and chi-square tests. First, PCA with Varimax rotation was conducted to identify the underlying structure of the selected food-related attitudes and choice criteria. The retained components were subsequently used in a two-step cluster analysis to identify consumer segments. Two-step cluster analysis was selected because it uses log-likelihood distance measures in combination with BIC-based automatic model selection, which is well-suited to segmentation variables measured at different scale levels and objectifies the cluster selection process without arbitrary researcher intervention [41]. Unlike hierarchical agglomerative methods, it processes larger survey samples efficiently through a pre-clustering phase that builds a cluster features tree, ensuring high scalability [42]. Compared with k-means clustering, the two-step procedure does not require a single number of clusters to be specified a priori, but allows for alternative solutions to be evaluated within an automatic clustering framework [41,42]. Alternative cluster solutions ranging from two to five clusters were examined using the Bayesian Information Criterion based on the log-likelihood distance measure (BIC-LL) and the distance measure ratio [18,43]. The automatic BIC-based selection criterion identified the four-cluster solution as optimal; however, the five-cluster solution was retained as the final solution on the basis of substantive interpretability, as it provided a more theoretically meaningful differentiation of consumer segments consistent with the conceptual framework of the study [44]. The silhouette measure of cohesion and separation for the five-cluster solution indicated fair cluster quality. Cluster sizes ranged from 243 (16.1%) to 349 (23.2%) respondents, with a largest-to-smallest size ratio of 1.42. After the clusters were identified, they were profiled using socio-demographic characteristics, health-oriented choice criteria, and frequency of food consumption variables. Differences between clusters in categorical variables, including socio-demographic characteristics and consumption-frequency categories, were examined using Pearson’s chi-square tests. Differences in mean scores for continuous or Likert-type profiling variables were tested using one-way ANOVA. When the overall F test was statistically significant, post hoc pairwise comparisons between clusters were performed using Scheffé tests. In the tables, different superscript letters within a row indicate statistically significant differences between cluster means at *p* < 0.05. All analyses were conducted using IBM SPSS Statistics, version 31.0 (IBM Corp., Armonk, NY, USA).

### 2.4. Characteristics of the Sample

The sample included 1508 respondents, evenly split between women (50%) and men (50%). The largest age group was 35–44 years old (25.7%), followed by respondents aged 45–54 years (22.7%), 55–70 years (20.8%), 25–34 years (19.8%) and 18–24 years (10.9%). In terms of education, 39.0% of respondents had secondary education, 31.0% had higher education, and 30.0% had primary or vocational education. The sample included both rural and urban consumers: 38.0% lived in rural areas, while 21.0% lived in cities with populations of 200,000 or more. Most respondents lived in two-, three-, or four-person households, and 47.0% reported having children under 18 years of age in the household. The dominant self-assessed financial situation was moderate: 60.7% reported that their household had enough money for basic needs but needed to save for larger expenses.

## 3. Results

### 3.1. Results of Factor Analysis of Segmenting Variables

Principal component analysis (PCA) with Varimax rotation was conducted to identify the underlying structure of food-related attitudes and choice criteria. The data were suitable for factor analysis, as indicated by a high Kaiser–Meyer–Olkin measure of sampling adequacy (KMO = 0.892) and a statistically significant Bartlett’s test of sphericity (χ^2^(325) = 18,243.163, *p* < 0.001). Six components with eigenvalues greater than 1 were retained, together explaining 65.0% of the total variance. Internal consistency was assessed using Cronbach’s alpha for each component. The reliability coefficients indicated satisfactory to good internal consistency, supporting the use of the retained components as internally consistent empirical dimensions of food-related attitudes and choice criteria in the subsequent cluster analysis. The first component, labelled sustainable and ethical choices, comprised items related to quality and sustainability certification, regional certification, regional food, organic food, environmentally friendly production, free-range eggs, avoidance of eggs from caged hens, and fish from sustainable fisheries (Table 1).

This component showed good internal consistency (α = 0.862). The second component, labelled meat reduction, included items referring to using legumes to reduce meat consumption, replacing meat with legumes, avoiding meat, and choosing foods that contain plant proteins. It captured a dietary orientation toward reducing meat intake and incorporating plant-based protein sources into everyday eating practices. Internal consistency was good (α = 0.848). The third component was labelled food involvement, consistent with the original FRL measurement instrument. This component represented affective, experiential, and lifestyle-related engagement with food and showed good internal consistency (α = 0.835). The fourth component, labelled importance of product information, also reflected the FRL framework. It consisted of items describing the importance of package information, comparing labels to select products with higher nutritional value, and comparing product information before making a choice. This component represented an information-oriented approach to food choice, in which consumers actively use product labels to support evaluation and decision-making. Internal consistency was good (α = 0.835). The fifth component, labelled shopping list, comprised items from FRL referring to preparing a shopping list before buying food, using the list during shopping, and the tendency to buy a few more things than planned. It showed acceptable to good internal consistency (α = 0.774). The sixth component, labelled food-waste avoidance, included items from SHE related to not wasting food, using leftovers to prepare new dishes or snacks, and avoiding throwing food away. It reflected household practices aimed at reducing food waste and showed acceptable internal consistency (α = 0.735).

Following the principal component analysis, component scores were used as input variables in the cluster analysis. The resulting five-cluster classification was then described in terms of segment size and respondents’ socio-demographic characteristics. The clusters were relatively comparable in size. Cluster 1 and Cluster 5 were the largest, each accounting for 23.2% of the sample, followed by Cluster 4 (19.9%), Cluster 3 (17.7%) and Cluster 2 (16.1%).

### 3.2. Segment Description

The five clusters did not differ significantly by gender, χ^2^(4) = 5.453, *p* = 0.244; place of residence, χ^2^(16) = 18.668, *p* = 0.286; or self-assessed financial situation, χ^2^(20) = 24.908, *p* = 0.205. Significant differences were observed for age, χ^2^(16) = 86.796, *p* < 0.001; education, χ^2^(8) = 27.686, *p* < 0.001; household size, χ^2^(36) = 85.072, Monte Carlo *p* < 0.001; and the presence of children under 18 in the household, χ^2^(4) = 23.845, *p* < 0.001 (Table 2). Given the non-significant differences in gender, place of residence, and financial situation, the socio-demographic interpretation focused mainly on age, education, and household composition.

Cluster 1 closely resembled the total sample in terms of the main socio-demographic characteristics. Women accounted for a slightly higher share of this cluster (53.0%), although gender differences across clusters were not statistically significant. The age distribution was relatively balanced, and almost half of respondents lived in households with children under 18. Cluster 2 also showed a broadly balanced socio-demographic composition, with a slightly higher share of respondents with higher education and households with children. Cluster 3 was the oldest segment and was more often composed of respondents without children under 18, with a relatively high proportion of two-person households and higher educational attainment. Cluster 4 was less socio-demographically distinctive, although it included a somewhat higher share of middle-aged respondents. Cluster 5 was the youngest segment and was most likely to include households with children under 18; it also had the highest share of respondents with primary or vocational education. Although Cluster 5 contained a slightly higher proportion of respondents reporting financial difficulties, self-assessed financial situation did not significantly differentiate the clusters.

### 3.3. Consumer Segment Profiles Based on Food-Related Attitudes and Choice Criteria

Across the total sample, mean scores were highest for selected items related to food involvement and food-waste avoidance (Table 3). The highest values were observed for avoiding food waste, loving good food, and perceiving food and drink as an important part of life. Mean scores for label-use items were also relatively high, particularly for the importance attached to product information. Sustainability-related criteria showed a more differentiated pattern, with moderate mean scores for regional food, environmentally friendly production, and certificates or quality marks, and lower mean scores for meat avoidance and the use of legumes as meat-substitutes.

On the basis of differences in the segmentation-variable patterns, the five clusters were labelled as follows: Sustainability and meat-reduction orientation (Cluster 1), High involvement and label/quality attention (Cluster 2), Moderate conventional orientation with stronger food-waste avoidance (Cluster 3), Hedonic orientation with lower sustainability emphasis (Cluster 4), and Low engagement with selective meat-reduction tendencies (Cluster 5). These labels should be understood as interpretive descriptors of the dominant tendencies observed within each cluster. Cluster 1 showed the most consistently sustainability- and meat-reduction-oriented pattern, with high scores for sustainability-related product criteria, animal-welfare-related criteria, meat reduction, and plant-protein substitution. Cluster 2 was characterised by relatively high food involvement and greater attention to labels, quality cues and product information. However, unlike Cluster 1, this group was not strongly oriented toward meat avoidance or the replacement of meat with legumes. Cluster 2, therefore, represents highly food-involved consumers who are attentive to labels, quality cues and product information, but less committed to meat reduction. Cluster 3 displayed a moderate and comparatively conventional profile. Respondents in this cluster had relatively high mean scores for product-information importance, but lower scores for label comparison than Clusters 1 and 2. They also showed lower food involvement, especially regarding the pleasure, lifestyle, and social meanings of food. Their agreement with sustainability-related criteria was moderate, whereas their agreement with meat-reduction and plant-protein substitution criteria was weak. At the same time, this cluster had high mean scores for food-waste avoidance. Cluster 4 had the lowest or among the lowest mean scores for most label-use, sustainability-related, animal-welfare-related and meat-reduction indicators. However, food involvement in this cluster was not uniformly low: respondents still expressed a relatively strong appreciation of good food and regarded food and drink as an important part of life. Cluster 4 therefore represented a more hedonic profile combined with lower sustainability-related engagement. Cluster 5 was distinguished by lower food involvement, weaker shopping planning and food-waste avoidance, alongside selective meat-reduction tendencies that remained less pronounced than in Cluster 1.

### 3.4. Consumption Profiles of the Identified Consumer Clusters

Reported consumption frequency was compared across clusters for dairy products, PBDAs, and meat, fish and legume dishes. Table 4 presents the percentage of respondents reporting consumption at least once per week, combining the response categories “once/week”, “several times/week”, “once/day” and “several times/day”. Full consumption-frequency distributions are provided in Supplementary Tables S1–S3. Cluster differences in consumption frequency were statistically significant for most product categories. Within dairy products, significant differences were observed for milk (*p* = 0.004), fermented milk drinks (*p* < 0.001) and cottage/quark cheese (*p* < 0.001), whereas Gouda-type cheese did not significantly differentiate the clusters (*p* = 0.607). All PBDA categories differed significantly across clusters (*p* < 0.001). Significant differences were also found for red meat dishes (*p* = 0.002), white meat dishes, fish and legume dishes (all *p* < 0.001).

However, dairy products and meat remained present in this cluster, indicating a diversified dietary pattern rather than full replacement of animal-based products. Cluster 2 represented a relatively conventional consumption profile despite strong food involvement and label/quality attention. Respondents in this segment reported frequent consumption of dairy products, especially fermented milk drinks and cottage/quark cheese, as well as relatively frequent white meat and fish consumption. PBDA use was lower than in Cluster 1 and, for several categories, also lower than in Cluster 5, but generally higher than in Clusters 3 and 4. Legume dishes were most often consumed once a week or one to three times a month, indicating moderate rather than strongly substitution-oriented legume consumption. Clusters 3 and 4 showed the lowest PBDAs and legume consumption. In Cluster 3, low PBDAs use co-occurred with infrequent legume intake and a generally conventional pattern of dairy and meat consumption. Cluster 4 reported the least frequent consumption of PBDAs and legumes overall, alongside common white-meat consumption and relatively infrequent use of fermented milk drinks and cottage/quark cheese. This pattern suggests limited incorporation of plant-based dairy alternatives and legumes. Cluster 5 was characterised by a more selective consumption profile. Respondents in this segment consumed PBDAs more often than those in Clusters 3 and 4, but less consistently than respondents in Cluster 1. They also reported relatively frequent consumption of plant-based cheese alternatives and legume dishes compared with the more conventional clusters. Fish consumption was relatively frequent, particularly at several times per week or once per day. Dairy consumption was moderate, although this segment included relatively high proportions of respondents who never consumed fermented milk drinks or cottage/quark cheese.

### 3.5. Health-Oriented Choice Criteria Across Consumer Clusters

The health-oriented profiling variables further differentiated the clusters (Table 5). All analysed items differed significantly across clusters (*p* < 0.001). In the total sample, mean scores were highest for choosing foods that support health, choosing nutritious foods, and choosing foods rich in vitamins and minerals. Lower mean scores were observed for choosing foods without additives, avoiding sugar-sweetened beverages, and limiting salt intake, although these values remained above the scale midpoint.

Cluster 1 showed the most consistently health-oriented profile, with among the highest mean scores for choosing nutritious foods, health-supporting foods, foods rich in vitamins and minerals, and foods without additives. It was also clearly distinguished by the highest mean scores for avoiding sugar-sweetened beverages and limiting salt intake. Cluster 2 showed a differentiated pattern across the health-oriented criteria. Mean scores for choosing nutritious foods, foods that support health, and foods rich in vitamins and minerals were comparable to those observed in Cluster 1. The mean score for choosing foods without additives was also relatively high. By contrast, mean scores for avoiding sugar-sweetened beverages and limiting salt intake were lower than in Cluster 1 and similar to those observed in Cluster 3. This pattern indicates that Cluster 2 scored higher on general nutrition- and health-related criteria than on avoidance-oriented criteria. Cluster 3 occupied an intermediate position, with lower scores than Clusters 1 and 2 for general health-oriented criteria, but higher scores than Clusters 4 and 5. Clusters 4 and 5 had the lowest or among the lowest mean scores across the health-oriented criteria. Cluster 4 combined relatively low scores on health-oriented profiling variables with low scores on sustainability-related segmentation variables. In Cluster 5, a similarly less pronounced health-oriented profile was observed alongside relatively higher scores on selected meat-reduction items compared with the more conventional clusters. Thus, selective meat-reduction tendencies in this segment did not correspond to a broader health-oriented approach to food choice.

## 4. Discussion

### 4.1. Segment-Specific Co-Occurrence of Health, Sustainability and Dairy-Related Choice Patterns

The present study identified five consumer segments characterised by distinct food-related orientations, self-reported consumption patterns and health-related choice criteria. Because the study employed a cross-sectional design with retrospective, self-reported data, the patterns described below represent associations and co-occurrences between consumer characteristics and consumption behaviours, rather than directional or causal relationships. Within this framework, Cluster 1 showed a profile in which sustainability-related product criteria, a meat-reduction orientation, frequent consumption of legume dishes, and PBDA use tended to co-occur. In contrast, other segments showed more selective patterns; for example, relatively high food-waste avoidance was not accompanied by similarly frequent consumption of PBDAs or legume dishes. These findings reinforce the view that sustainability-related food behaviour is not organised along a single continuum from low to high sustainability [45,46]. Rather, product-related criteria, dietary change and household food-management practices appeared to represent partly distinct dimensions that converged in some segments but remained weakly connected in others. This interpretation aligns with previous segmentation research showing that sustainable food consumption is expressed through different behavioural strategies rather than through a single homogeneous pattern [28,47,48,49]. The results indicate that, in some segments, PBDAs and legume consumption tend to co-occur with broader patterns of dietary diversification. These patterns also have nutritional implications. PBDAs differ substantially in nutritional composition across product types and formulations. Soy-based alternatives are generally closer to dairy milk in protein content, whereas oat-, almond-, rice- and other plant-based alternatives often provide lower amounts of protein and may differ in key micronutrients, including calcium, vitamin B12, iodine and vitamin B2, unless they are appropriately fortified [23,24,25]. Therefore, the nutritional relevance of PBDA consumption depends not only on the frequency of use, but also on the type of products selected, their fortification status and the extent to which they replace dairy products in the diet. Although nutrient intake was not assessed directly, the pattern observed in Cluster 1 is relevant from a nutritional perspective because more frequent reported consumption of PBDAs co-occurred with more frequent reported consumption of legume dishes and stronger meat-reduction orientation. These product groups have different nutritional implications. PBDAs are relevant in the context of potential dairy replacement, particularly because their nutritional value depends on product type, formulation and fortification status. Legume dishes, in turn, contribute to plant-protein diversification and provide dietary fibre, B vitamins, iron and zinc, which makes them relevant in dietary patterns oriented toward reducing meat consumption [50,51]. However, more frequent reported consumption of PBDAs cannot be assumed to provide nutritional equivalence with dairy products, particularly with respect to calcium, iodine, vitamin B12 and protein, unless fortified products with appropriate nutrient profiles are selected [23,25]. These findings suggest that dietary guidance should address not only the sustainability dimensions of plant-based dietary diversification, but also the nutritional adequacy of resulting dietary patterns. Such guidance may be particularly relevant for consumer segments in which consumption of PBDAs forms part of broader plant-based dietary diversification. The profile of Cluster 2, characterised by high involvement and label/quality attention, points to an important distinction between food involvement, health-oriented quality evaluation and dietary substitution. Respondents in this segment showed the highest food involvement and the strongest reliance on product information and labels. They also scored highly on general health-related criteria. However, this profile was not accompanied by strong meat-reduction orientation or frequent PBDA consumption. In this segment, cognitive engagement with food appeared to focus mainly on product quality, nutritional value and health-related attributes, rather than on meat reduction or more frequent PBDA consumption [28,52,53]. Thus, food involvement and health-oriented criteria may support reflective, quality-oriented food choices, but do not necessarily co-occur with meat-reduction orientation or frequent PBDA consumption. This suggests that health- and quality-related considerations alone may be insufficient to support meat-reduction or PBDA-oriented patterns; these may require stronger sustainability- or ethics-related orientations [53,54]. Animal-welfare-related criteria add a further distinction to the interpretation of the segments. In the present study, animal welfare was relevant not only in the segment most strongly oriented toward meat reduction, but also in the cluster characterised by high involvement and label/quality attention. This suggests that animal welfare may act both as part of a meat-reduction-oriented profile and as a perceived quality and ethical cue within otherwise conventional dairy and meat consumption patterns. This interpretation is consistent with research showing that animal welfare can be a salient attribute in meat and dairy choices, including among consumers who do not necessarily reject animal-based foods [34].

### 4.2. Selective Sustainability Related Patterns and Segment Specific PBDA Use

The remaining clusters indicate that sustainability-related food behaviour was selective and segment-specific rather than uniformly organised around PBDA consumption, legume-dish consumption, or meat reduction. Cluster 3 illustrates a responsibility-oriented food practice centred on household food management rather than on product choice or dietary substitution. This aligns with the literature showing that household food waste is shaped by planning and shopping routines, food-management practices and multiple motivational bundles, including practical, economic, environmental and emotional considerations [36,55]. Respondents in this segment reported relatively conventional consumption patterns, with limited PBDA and legume-dish consumption but stronger food-waste avoidance. Food-waste avoidance is sustainability-relevant, but it may not necessarily co-occur with product-related sustainability criteria or substitution-oriented dietary change [56,57]. Thus, Cluster 3 should not be interpreted simply as low in sustainability engagement, but as representing a food-management-focused pattern in which responsibility was more strongly expressed through avoiding food waste than through dietary substitution. The profile of Cluster 4, characterised by hedonic orientation with lower sustainability emphasis, illustrates that food appreciation may be expressed primarily through pleasure and enjoyment, without being accompanied by stronger reflective or responsibility-oriented food-choice criteria. Respondents in this segment valued good food and regarded food and drink as an important part of life, but reported low engagement with label comparison, sustainability-related criteria, animal-welfare-related criteria, and meat-reduction indicators. They also reported the least frequent consumption of plant-based dairy alternatives. This pattern indicates that the affective and pleasure-related dimension of food involvement did not coincide with a more information-oriented, health-oriented or sustainability-oriented approach to food choice. In this segment, culinary enjoyment appeared to remain largely separate from criteria related to product information, ethical attributes and dietary substitution. Cluster 5 further indicates that PBDA consumption is not necessarily embedded in a broader health- or sustainability-oriented food-choice profile [9,58]. Although respondents in this segment reported more frequent consumption of some PBDAs and legume dishes than respondents in the more conventional clusters, they also exhibited relatively low food involvement, low food-waste avoidance, and low health-oriented scores. The data do not allow for direct conclusions about the motives underlying PBDA or legume-dish consumption in Cluster 5. However, the relatively frequent reported consumption of legume dishes in Cluster 5 was not accompanied by high food involvement or stronger health-oriented scores. This pattern differed from Cluster 1, where legume-dish consumption co-occurred with stronger sustainability and meat-reduction orientation. In Cluster 5, it appeared as a more selective element of dietary diversification. This interpretation is consistent with research showing that legume consumption is shaped by heterogeneous factors, including health, sensory preferences, familiarity, preparation, price and convenience [59]. The characteristic of Cluster 5 suggests that specific meat-reduction or plant-based behaviours can occur without forming part of a comprehensive health-, sustainability- or ethics-oriented food-choice framework [54]. These tendencies coexisted with lower food involvement, weaker food-waste avoidance and less pronounced health-oriented criteria, indicating a selective rather than consistently developed pattern of plant-based dietary diversification. Given that this segment was also younger and included the highest proportion of households with children under 18, one hypothesis is that the incorporation of PBDAs or legumes may reflect pragmatic forms of dietary diversification, potentially related to household preferences, convenience, product availability, sensory curiosity, or routine meal variation rather than a coherent sustainability-oriented dietary strategy [60,61]. Cluster 5 shows that selected plant-based options can be incorporated without forming part of a coherent health-, sustainability- or ethics-oriented food-choice profile, as these tendencies coexisted with lower food involvement, weaker food-waste avoidance and less pronounced health-oriented criteria.

### 4.3. Limitations and Future Research

This study has several limitations. First, the study was cross-sectional and based on self-reported survey data, including retrospective reports of food-consumption frequency. Therefore, the identified segments should be interpreted as patterns of co-occurrence among food-related orientations, health-oriented choice criteria and reported consumption frequencies, rather than as evidence of causal relationships. The data do not allow for conclusions about whether food involvement, sustainability orientation or health-related criteria precede, result from, or explain differences in reported consumption of dairy products or plant-based dairy alternatives. Second, although the sample was structured to reflect Polish adults in terms of key socio-demographic characteristics, respondents were recruited from an online access panel and were eligible only if they were responsible or co-responsible for household food purchasing. This recruitment mode may introduce self-selection bias, as individuals participating in online survey panels may differ from the broader population in terms of digital access, survey participation propensity, food-related interests, health consciousness or sustainability awareness. The findings should therefore be generalised with caution. Third, the segmentation variables were based on selected items adapted from established instruments rather than on complete validated scales. Consequently, the retained PCA components should be understood as study-specific empirical dimensions rather than direct replications of the original constructs. Fourth, the cluster solution should be interpreted as exploratory. Alternative cluster solutions were examined, and the five-cluster solution was retained on the basis of statistical diagnostics and substantive interpretability. However, the silhouette measure indicated fair cluster quality, suggesting that some overlap between clusters should be expected. Therefore, the identified clusters should not be treated as naturally bounded or fully homogeneous consumer groups. The cluster labels used in this study are researcher-assigned interpretive descriptors of dominant tendencies within statistically derived groups. Future studies could assess the robustness of these patterns using independent samples, holdout validation procedures or alternative segmentation methods, such as latent class analysis or model-based clustering. Fifth, the food-consumption data were based on frequency categories and did not include portion sizes, nutrient intake, dietary adequacy or detailed product-level information. As a result, the study cannot determine the nutritional implications of dairy or PBDA consumption in individual diets. This is particularly relevant because PBDAs differ by product type, base ingredient, fortification status and nutrient composition. Future research should combine consumption-frequency data with dietary intake assessment and product-level nutrient-composition data to evaluate implications for protein, calcium, iodine, vitamin B12 and other nutrients of concern. Finally, the study focused on Polish consumers responsible or co-responsible for household food purchasing. Cultural, market and regulatory contexts may influence both dairy consumption and the adoption of plant-based alternatives. Cross-national studies would therefore be valuable for examining whether similar food-related orientations and consumption patterns emerge under different dietary, market and regulatory conditions.

## 5. Conclusions

The findings indicate that reported PBDA consumption is segment-dependent and should be interpreted in relation to segment-specific configurations of food-related orientations and everyday practices, rather than as evidence of a simple replacement of dairy products. The identified segments show that dairy and PBDA consumption were embedded in broader patterns of food involvement, product-information use, sustainability-related priorities, meat-reduction orientation, food-waste avoidance and health-oriented choice criteria. The most consistent sustainability- and meat-reduction-oriented pattern was observed in Cluster 1, where environmental and animal-welfare criteria, meat-reduction orientation, more frequent PBDA consumption and more frequent consumption of legume dishes co-occurred. Nevertheless, dairy products and meat remained part of the reported diet in this segment, indicating dietary diversification rather than complete substitution of animal-derived foods. The remaining segments show that sustainability-relevant food behaviour was not organised along a single continuum, but reflected distinct product-related, dietary, and household-management dimensions. PBDA consumption should therefore not be interpreted as a stand-alone indicator of a sustainable or health-oriented dietary pattern. Its interpretation depends on the broader segment profile: in some segments, PBDA consumption co-occurred with sustainability-related criteria, meat-reduction orientation and more frequent legume-dish consumption, whereas in others it reflected a more selective dietary diversification within otherwise conventional consumption patterns.

## Figures and Tables

**Table 1:** The results of factor analysis on the segmenting variables.

Item	Factor 1	Factor 2	Factor 3	Factor 4	Factor 5	Factor 6
To me product information is of major importance. I want to know what the product contains.				0.762		
I compare labels to select the most nutritious food.				0.763		
I compare product information labels to decide which brand to try.				0.816		
Before I go shopping for food, I make a list of everything I need.					0.852	
I make a shopping list to guide my food purchases.					0.848	
I have a tendency to buy a few more things than I had planned.					0.682	
I just love good food.			0.794			
Eating and drinking are a continuous source of joy for me.			0.835			
Decisions on what to eat and drink are very important to me.			0.508			
Food and drink is an important part of my life.			0.821			
Eating and food is an important part of my social life.			0.765			
When buying food, I check certificates and quality marks on labels.	0.544					
I choose products with a regional certificate.	0.608					
I choose food produced in an environmentally friendly way.	0.564					
I buy regional food.	0.615					
Whenever possible, I buy organic food.	0.575					
I try to eat as many legumes as possible to reduce meat consumption.		0.848				
Legumes replace meat in my kitchen.		0.846				
I avoid eating meat.		0.784				
I try to eat as many food products containing plant proteins, e.g., legumes, as possible.		0.663				
I do not waste food.						0.845
I reuse leftovers from food.						0.608
I try not to throw food away.						0.850
I choose eggs from free-range hens.	0.770					
I avoid buying eggs from caged hens.	0.737					
Whenever possible, I buy fish from sustainable fisheries.	0.557					

Note: The item “I have a tendency to buy a few more things than I had planned” was reverse-coded before PCA.

**Table 2:** Socio-demographic characteristics of the total sample and by cluster.

Characteristic	Total	Cluster 1	Cluster 2	Cluster 3	Cluster 4	Cluster 5
Gender						
Male	754 (50.0%)	164 (47.0%)	123 (50.6%)	123 (46.1%)	161 (53.8%)	183 (52.3%)
Female	754 (50.0%)	185 (53.0%)	120 (49.4%)	144 (53.9%)	138 (46.2%)	167 (47.7%)
Age						
18–24	165 (10.9%)	30 (8.6%)	29 (12.0%)	15 (5.6%)	41 (13.7%)	50 (14.3%)
25–34	299 (19.8%)	70 (20.0%)	48 (19.8%)	32 (12.0%)	50 (16.7%)	99 (28.4%)
35–44	388 (25.7%)	90 (25.7%)	66 (27.3%)	56 (21.0%)	78 (26.0%)	98 (28.1%)
45–54	343 (22.8%)	82 (23.4%)	55 (22.7%)	72 (27.0%)	72 (24.0%)	62 (17.8%)
55–70	314 (20.8%)	78 (22.3%)	44 (18.2%)	92 (34.5%)	59 (19.7%)	40 (11.5%)
Education						
Primary/vocational	452 (30.0%)	108 (30.9%)	60 (24.8%)	58 (21.7%)	88 (29.3%)	138 (39.5%)
Secondary	588 (39.0%)	139 (39.8%)	101 (41.7%)	116 (43.4%)	116 (38.7%)	116 (33.2%)
Higher	467 (31.0%)	102 (29.2%)	81 (33.5%)	93 (34.8%)	96 (32.0%)	95 (27.2%)
Place of residence						
Rural area	573 (38.0%)	134 (38.3%)	86 (35.4%)	99 (37.2%)	113 (37.8%)	141 (40.4%)
Town up to 20,000	196 (13.0%)	46 (13.1%)	34 (14.0%)	40 (15.0%)	30 (10.0%)	45 (12.9%)
Town 20,000–50,000	167 (11.1%)	47 (13.4%)	31 (12.8%)	25 (9.4%)	30 (10.0%)	34 (9.7%)
City 50,000–200,000	256 (17.0%)	59 (16.9%)	36 (14.8%)	56 (21.1%)	48 (16.1%)	57 (16.3%)
City above 200,000	316 (21.0%)	64 (18.3%)	56 (23.0%)	46 (17.3%)	78 (26.1%)	72 (20.6%)
Household size						
1	156 (10.4%)	36 (10.3%)	18 (7.4%)	26 (9.7%)	41 (13.7%)	35 (10.0%)
2	419 (27.8%)	86 (24.6%)	68 (28.1%)	98 (36.7%)	84 (28.0%)	83 (23.8%)
3	388 (25.7%)	91 (26.1%)	48 (19.8%)	60 (22.5%)	88 (29.3%)	101 (28.9%)
4	360 (23.9%)	96 (27.5%)	69 (28.5%)	56 (21.0%)	59 (19.7%)	80 (22.9%)
Above 5	69 (4.6%)	11 (3.2%)	23 (9.5%)	3 (1.1%)	11 (3.7%)	21 (6.0%)
Children < 18 in household						
Yes	636 (47.0%)	155 (49.5%)	115 (51.1%)	85 (35.3%)	111 (42.9%)	170 (54.1%)
No	716 (53.0%)	158 (50.5%)	110 (48.9%)	156 (64.7%)	148 (57.1%)	144 (45.9%)
Self-assessed financial situation						
We live very poorly–insufficient even for basic needs	43 (2.9%)	10 (2.9%)	2 (0.8%)	5 (1.9%)	10 (3.3%)	16 (4.6%)
We live modestly–we must economise a lot	162 (10.7%)	44 (12.6%)	16 (6.6%)	30 (11.3%)	30 (10.0%)	42 (12.0%)
We live averagely–enough for daily needs; must save for bigger purchases	915 (60.7%)	199 (57.0%)	152 (62.8%)	161 (60.5%)	191 (63.5%)	211 (60.5%)
We live well–without significant savings	309 (20.5%)	78 (22.3%)	60 (24.8%)	59 (22.2%)	54 (17.9%)	58 (16.6%)
We live very well–we can afford some luxuries	22 (1.5%)	7 (2.0%)	3 (1.2%)	3 (1.1%)	5 (1.7%)	5 (1.4%)
Difficult to say	56 (3.7%)	11 (3.2%)	9 (3.7%)	8 (3.0%)	11 (3.7%)	17 (4.9%)

Values are presented as *n* (% within cluster). Percentages are based on valid responses and may not sum to 100% because of rounding and missing responses.

**Table 3:** Food involvement, label use, and sustainability-oriented choice criteria across consumer clusters.

Items	Total	Cluster 1	Cluster 2	Cluster 3	Cluster 4	Cluster 5
To me product information is of major importance. I want to know what the product contains.	5.19	5.58 ^b^	6.30 ^c^	5.61 ^b^	4.28 ^a^	4.50 ^a^
I compare labels to select the most nutritious food.	4.60	5.25 ^c^	5.62 ^d^	4.56 ^b^	3.40 ^a^	4.28 ^b^
I compare product information labels to decide which brand to try.	4.72	5.25 ^d^	5.81 ^e^	4.88 ^c^	3.53 ^a^	4.34 ^b^
Before I go shopping for food, I make a list of everything I need.	5.07	5.39 ^b^	5.06 ^b^	5.23 ^b^	5.06 ^b^	4.63 ^a^
I make a shopping list to guide my food purchases.	5.13	5.52 ^c^	5.18 ^bc^	5.25 ^bc^	5.09 ^b^	4.66 ^a^
I have a tendency to buy a few more things than I had planned.	4.04	3.67 ^a^	4.05 ^ab^	4.59 ^c^	4.23 ^bc^	3.84 ^ab^
I just love good food.	5.48	5.56 ^b^	6.55 ^c^	4.87 ^a^	5.76 ^b^	4.87 ^a^
Eating and drinking are a continuous source of joy for me.	5.04	5.37 ^c^	6.17 ^d^	4.08 ^a^	5.18 ^c^	4.53 ^b^
Decisions on what to eat and drink are very important to me.	5.21	5.61 ^c^	6.23 ^d^	5.02 ^b^	4.80 ^ab^	4.60 ^a^
Food and drink is an important part of my life	.5.43	5.49 ^b^	6.38 ^c^	4.58 ^a^	5.60 ^b^	4.84 ^a^
Eating and food is an important part of my social life.	4.76	5.16 ^d^	5.71 ^e^	3.80 ^a^	4.79 ^c^	4.40 ^b^
When buying food, I check certificates and quality marks on labels.	4.31	5.42 ^e^	5.06 ^d^	4.53 ^c^	2.66 ^a^	3.94 ^b^
I choose products with a regional certificate.	4.31	5.37 ^e^	4.95 ^d^	4.42 ^c^	2.88 ^a^	3.97 ^b^
I choose food produced in an environmentally friendly way.	4.59	5.59 ^e^	5.10 ^d^	4.65 ^c^	3.48 ^a^	4.12 ^b^
I buy regional food.	4.77	5.59 ^d^	5.36 ^d^	4.98 ^c^	3.79 ^a^	4.22 ^b^
Whenever possible, I buy organic food.	4.24	5.36 ^e^	4.86 ^d^	4.39 ^c^	2.66 ^a^	3.94 ^b^
I try to eat as many legumes as possible to reduce meat consumption.	3.71	5.70 ^d^	3.14 ^b^	2.89 ^ab^	2.70 ^a^	3.60 ^c^
Legumes replace meat in my kitchen.	3.31	5.37 ^d^	2.79 ^b^	2.17 ^a^	2.18 ^a^	3.43 ^c^
I avoid eating meat.	3.16	4.92 ^c^	2.30 ^a^	2.48 ^a^	2.13 ^a^	3.41 ^b^
I try to eat as many plant-protein foods as possible, e.g., legumes.	4.24	5.61 ^d^	4.37 ^c^	3.74 ^ab^	3.47 ^a^	3.84 ^b^
I do not waste food.	5.62	6.21 ^c^	5.90 ^b^	6.12 ^bc^	5.93 ^bc^	4.17 ^a^
I reuse leftovers from food.	5.01	5.70 ^c^	5.37 ^bc^	5.18 ^b^	5.03 ^b^	3.91 ^a^
I try not to throw food away.	5.88	6.36 ^b^	6.43 ^b^	6.53 ^b^	6.33 ^b^	4.16 ^a^
I choose free-range eggs.	5.42	5.88 ^b^	6.15 ^b^	5.99 ^b^	4.63 ^a^	4.70 ^a^
I avoid buying eggs from caged hens.	4.92	5.66 ^c^	5.35 ^c^	5.49 ^c^	3.89 ^a^	4.32 ^b^
Whenever possible, I buy fish from sustainable fishing.	4.28	5.30 ^d^	5.04 ^d^	4.40 ^c^	2.93 ^a^	3.78 ^b^

Values represent mean scores on a seven-point scale, anchored at “totally disagree” and “totally agree”. Different letters within rows indicate statistically significant differences between groups at *p* < 0.05 based on one-way ANOVA with Scheffé post hoc tests.

**Table 4:** Weekly or more frequent consumption of dairy products, plant-based dairy alternatives, and meat, fish, and legume dishes across clusters.

Product Category	Total	Cluster 1	Cluster 2	Cluster 3	Cluster 4	Cluster 5
Milk	1115 (74.0%)	273 (78.2%)	180 (74.4%)	189 (71.1%)	220 (73.6%)	253 (72.3%)
Fermented milk drinks	740 (49.1%)	227 (65.0%)	143 (58.8%)	119 (44.7%)	114 (38.0%)	137 (39.3%)
Cottage/quark cheese	1041 (69.0%)	281 (80.5%)	186 (76.9%)	168 (62.7%)	183 (61.0%)	223 (63.7%)
Gouda-type cheese	1262 (83.7%)	298 (85.1%)	213 (87.3%)	220 (83.0%)	253 (84.6%)	278 (79.4%)
Plant-based milk alternatives	358 (23.7%)	143 (41.0%)	60 (24.7%)	32 (12.0%)	27 (9.0%)	96 (27.4%)
Plant-based yoghurt/kefir alternatives	318 (21.1%)	137 (39.3%)	42 (17.3%)	22 (8.3%)	23 (7.7%)	94 (26.9%)
Plant-based cheese alternatives	245 (16.2%)	116 (33.1%)	28 (11.6%)	13 (4.9%)	12 (4.0%)	76 (21.8%)
Red meat dishes	991 (65.8%)	225 (64.5%)	172 (71.4%)	176 (66.2%)	186 (62.0%)	232 (66.5%)
White meat dishes	1265 (83.8%)	276 (78.9%)	218 (89.7%)	225 (84.6%)	259 (86.0%)	287 (82.0%)
Fish	879 (58.3%)	255 (73.1%)	161 (66.0%)	128 (47.9%)	126 (42.0%)	209 (59.9%)
Legume dishes	763 (50.5%)	281 (80.3%)	125 (51.4%)	80 (29.9%)	90 (30.0%)	187 (53.6%)

Values are presented as *n* (% within product-specific valid responses in each cluster) for respondents reporting consumption at least once per week. This category combines responses of once/week, several times/week, once/day and several times/day. Full consumption-frequency distributions are provided in Supplementary Tables S1–S3. Cluster 1 was distinguished by the most frequently reported consumption of all PBDA categories and legume dishes.

**Table 5:** Nutrition- and health-related choice criteria across clusters.

Item	Total	Cluster 1	Cluster 2	Cluster 3	Cluster 4	Cluster 5
I choose food that is nutritious.	5.09	5.79 ^c^	5.76 ^c^	5.20 ^b^	4.48 ^a^	4.36 ^a^
I choose foods that keep me healthy.	5.15	5.81 ^c^	5.86 ^c^	5.30 ^b^	4.48 ^a^	4.44 ^a^
I avoid sugar-sweetened beverages.	4.74	5.74 ^c^	4.78 ^b^	4.88 ^b^	3.96 ^a^	4.26 ^a^
I limit my salt intake.	4.52	5.63 ^c^	4.49 ^b^	4.53 ^b^	3.76 ^a^	4.10 ^a^
I choose food that contains a lot of vitamins and minerals.	5.03	5.73 ^c^	5.72 ^c^	5.12 ^b^	4.35 ^a^	4.38 ^a^
I choose foods that contain no additives.	4.91	5.69 ^c^	5.52 ^bc^	5.22 ^b^	4.07 ^a^	4.19 ^a^

Values represent mean scores on a seven-point scale, anchored at “totally disagree” and “totally agree”. Different letters within rows indicate statistically significant differences between groups at *p* < 0.05 based on one-way ANOVA with Scheffé post hoc tests.

## Data Availability

The data presented in this study are available on request from the corresponding author due to the data have not yet been made available in public databases.

## Supplementary Materials

The following supporting information can be downloaded at https://www.mdpi.com/article/10.3390/nu18132135/s1: Table S1. Consumption frequency of dairy products across clusters; Table S2. Consumption frequency of plant-based dairy alternatives across clusters; Table S3. Consumption frequency of meat, fish and legume dishes across clusters.
