# Supplementary material for: Dairy and Plant-Based Dairy Alternative Consumption Across Food-Related Consumer Segments: Food Involvement, Sustainability Orientation, and Health-Oriented Profiling
Source: Nutrients. 2026 Jul 2;18(13):2135. doi: 10.3390/nu18132135 (PMC13364209; doi:10.3390/nu18132135)
Supplement: Supplementary file 1 [file nutrients-18-02135-s001.zip › nutrients-4379273-supplementary.pdf]

Supplementary Table S1. Consumption frequency of dairy products across clusters.

Product and consumption frequency	Total n (%)	Cluster 1	Cluster 2	Cluster 3	Cluster 4	Cluster 5
Milk						
Never	116 (7.7%)	21 (6.0%)	15 (6.2%)	27 (10.2%)	24 (8.0%)	29 (8.3%)
1–3 times/month or less	275 (18.3%)	55 (15.8%)	47 (19.4%)	50 (18.8%)	55 (18.4%)	68 (19.4%)
Once/week	242 (16.1%)	63 (18.1%)	24 (9.9%)	28 (10.5%)	49 (16.4%)	78 (22.3%)
Several times/week	368 (24.4%)	89 (25.5%)	60 (24.8%)	72 (27.1%)	69 (23.1%)	78 (22.3%)
Once/day	297 (19.7%)	79 (22.6%)	57 (23.6%)	48 (18.0%)	63 (21.1%)	50 (14.3%)
Several times/day	208 (13.8%)	42 (12.0%)	39 (16.1%)	41 (15.4%)	39 (13.0%)	47 (13.4%)
Fermented milk drinks						
Never	240 (15.9%)	43 (12.3%)	24 (9.9%)	31 (11.7%)	68 (22.7%)	74 (21.2%)
1–3 times/month or less	527 (35.0%)	79 (22.6%)	76 (31.3%)	116 (43.6%)	118 (39.3%)	138 (39.5%)
Once/week	325 (21.6%)	91 (26.1%)	59 (24.3%)	52 (19.5%)	58 (19.3%)	65 (18.6%)
Several times/week	322 (21.4%)	103 (29.5%)	61 (25.1%)	55 (20.7%)	43 (14.3%)	60 (17.2%)
Once/day	76 (5.0%)	26 (7.4%)	20 (8.2%)	11 (4.1%)	10 (3.3%)	9 (2.6%)
Several times/day	17 (1.1%)	7 (2.0%)	3 (1.2%)	1 (0.4%)	3 (1.0%)	3 (0.9%)
Cottage/quark cheese						
Never	49 (3.2%)	7 (2.0%)	4 (1.7%)	8 (3.0%)	11 (3.7%)	19 (5.4%)
1–3 times/month or less	419 (27.8%)	61 (17.5%)	52 (21.5%)	92 (34.3%)	106 (35.3%)	108 (30.9%)
Once/week	432 (28.6%)	114 (32.7%)	60 (24.8%)	76 (28.4%)	88 (29.3%)	94 (26.9%)
Several times/week	472 (31.3%)	124 (35.5%)	100 (41.3%)	73 (27.2%)	74 (24.7%)	101 (28.9%)
Once/day	105 (7.0%)	27 (7.7%)	23 (9.5%)	18 (6.7%)	16 (5.3%)	21 (6.0%)
Several times/day	32 (2.1%)	16 (4.6%)	3 (1.2%)	1 (0.4%)	5 (1.7%)	7 (2.0%)
Gouda-type cheese						
Never	24 (1.6%)	5 (1.4%)	4 (1.6%)	4 (1.5%)	2 (0.7%)	9 (2.6%)
1–3 times/month or less	222 (14.7%)	47 (13.4%)	27 (11.1%)	41 (15.5%)	44 (14.7%)	63 (18.0%)
Once/week	359 (23.8%)	82 (23.4%)	51 (20.9%)	64 (24.2%)	73 (24.4%)	89 (25.4%)
Several times/week	657 (43.6%)	155 (44.3%)	119 (48.8%)	108 (40.8%)	137 (45.8%)	138 (39.4%)
Once/day	184 (12.2%)	48 (13.7%)	30 (12.3%)	35 (13.2%)	33 (11.0%)	38 (10.9%)
Several times/day	62 (4.1%)	13 (3.7%)	13 (5.3%)	13 (4.9%)	10 (3.3%)	13 (3.7%)

Note: Values are presented as n (% within cluster). Cluster labels are as follows: C1 = Sustainability and meat-reduction orientation; C2 = High involvement and label/quality attention; C3 = Moderate conventional orientation with stronger food-waste avoidance; C4 = Hedonic orientation with lower sustainability emphasis; C5 = Low engagement with selective meat-reduction tendencies. The same cluster numbering is used in Tables 5 and 6. Differences across clusters were tested using Pearson's chi-square test.

Supplementary Table S2. Consumption frequency of plant-based dairy alternatives across clusters.

Product and consumption frequency	Total n (%)	Cluster 1	Cluster 2	Cluster 3	Cluster 4	Cluster 5
Plant-based milk alternatives						
Never	794 (52.7%)	106 (30.4%)	119 (49.0%)	166 (62.4%)	222 (74.0%)	181 (51.7%)
1–3 times/month or less	356 (23.6%)	100 (28.7%)	64 (26.3%)	68 (25.6%)	51 (17.0%)	73 (20.9%)
Once/week	133 (8.8%)	49 (14.0%)	24 (9.9%)	14 (5.3%)	9 (3.0%)	37 (10.6%)
Several times/week	155 (10.3%)	64 (18.3%)	27 (11.1%)	14 (5.3%)	10 (3.3%)	40 (11.4%)

Once/day	51 (3.4%)	22 (6.3%)	6 (2.5%)	3 (1.1%)	6 (2.0%)	14 (4.0%)
Several times/day	19 (1.3%)	8 (2.3%)	3 (1.2%)	1 (0.4%)	2 (0.7%)	5 (1.4%)
Plant-based yoghurts/kefir alternatives						
Never	880 (58.4%)	121 (34.7%)	134 (55.1%)	191 (71.8%)	232 (77.6%)	202 (57.7%)
1–3 times/month or less	309 (20.5%)	91 (26.1%)	67 (27.6%)	53 (19.9%)	44 (14.7%)	54 (15.4%)
Once/week	135 (9.0%)	57 (16.3%)	20 (8.2%)	11 (4.1%)	11 (3.7%)	36 (10.3%)
Several times/week	136 (9.0%)	56 (16.0%)	19 (7.8%)	8 (3.0%)	10 (3.3%)	43 (12.3%)
Once/day	36 (2.4%)	18 (5.2%)	3 (1.2%)	3 (1.1%)	1 (0.3%)	11 (3.1%)
Several times/day	11 (0.7%)	6 (1.7%)	0 (0.0%)	0 (0.0%)	1 (0.3%)	4 (1.1%)
Plant-based cheese alternatives						
Never	1007 (66.8%)	152 (43.4%)	159 (65.7%)	218 (81.6%)	256 (85.0%)	222 (63.8%)
1–3 times/month or less	256 (17.0%)	82 (23.4%)	55 (22.7%)	36 (13.5%)	33 (11.0%)	50 (14.4%)
Once/week	117 (7.8%)	62 (17.7%)	12 (5.0%)	6 (2.2%)	4 (1.3%)	33 (9.5%)
Several times/week	99 (6.6%)	39 (11.1%)	14 (5.8%)	7 (2.6%)	4 (1.3%)	35 (10.1%)
Once/day	23 (1.5%)	13 (3.7%)	1 (0.4%)	0 (0.0%)	4 (1.3%)	5 (1.4%)
Several times/day	6 (0.4%)	2 (0.6%)	1 (0.4%)	0 (0.0%)	0 (0.0%)	3 (0.9%)

Note: Values are presented as n (% within cluster). Differences across clusters were tested using Pearson's chi-square test.

Supplementary Table S3. Consumption frequency of meat, fish and legume dishes across clusters.

Product and consumption frequency	Total n (%)	Cluster 1	Cluster 2	Cluster 3	Cluster 4	Cluster 5
Red meat dishes						
Never	128 (8.5%)	48 (13.8%)	9 (3.7%)	13 (4.9%)	30 (10.0%)	28 (8.0%)
1–3 times/month or less	386 (25.6%)	76 (21.8%)	60 (24.9%)	77 (28.9%)	84 (28.0%)	89 (25.5%)
Once/week	393 (26.1%)	91 (26.1%)	69 (28.6%)	68 (25.6%)	74 (24.7%)	91 (26.1%)
Several times/week	484 (32.2%)	96 (27.5%)	91 (37.8%)	88 (33.1%)	95 (31.7%)	114 (32.7%)
Once/day	87 (5.8%)	29 (8.3%)	9 (3.7%)	17 (6.4%)	11 (3.7%)	21 (6.0%)
Several times/day	27 (1.8%)	9 (2.6%)	3 (1.2%)	3 (1.1%)	6 (2.0%)	6 (1.7%)
White meat dishes						
Never	50 (3.3%)	27 (7.7%)	2 (0.8%)	3 (1.1%)	9 (3.0%)	9 (2.6%)
1–3 times/month or less	195 (12.9%)	47 (13.4%)	23 (9.5%)	38 (14.3%)	33 (11.0%)	54 (15.4%)
Once/week	406 (26.9%)	95 (27.1%)	46 (18.9%)	82 (30.8%)	80 (26.6%)	103 (29.4%)
Several times/week	691 (45.8%)	119 (34.0%)	144 (59.3%)	128 (48.1%)	155 (51.5%)	145 (41.4%)
Once/day	126 (8.3%)	39 (11.1%)	22 (9.1%)	14 (5.3%)	19 (6.3%)	32 (9.1%)
Several times/day	42 (2.8%)	23 (6.6%)	6 (2.5%)	1 (0.4%)	5 (1.7%)	7 (2.0%)
Fish						
Never	74 (4.9%)	17 (4.9%)	5 (2.0%)	7 (2.6%)	22 (7.3%)	23 (6.6%)
1–3 times/month or less	556 (36.8%)	77 (22.1%)	78 (32.0%)	132 (49.4%)	152 (50.7%)	117 (33.5%)
Once/week	540 (35.8%)	130 (37.2%)	113 (46.3%)	100 (37.5%)	96 (32.0%)	101 (28.9%)
Several times/week	237 (15.7%)	76 (21.8%)	36 (14.8%)	23 (8.6%)	26 (8.7%)	76 (21.8%)
Once/day	66 (4.4%)	25 (7.2%)	8 (3.3%)	2 (0.7%)	3 (1.0%)	28 (8.0%)
Several times/day	36 (2.4%)	24 (6.9%)	4 (1.6%)	3 (1.1%)	1 (0.3%)	4 (1.1%)
Legume dishes						
Never	107 (7.1%)	4 (1.1%)	21 (8.6%)	25 (9.3%)	28 (9.3%)	29 (8.3%)
1–3 times/month or less	640 (42.4%)	65 (18.6%)	97 (39.9%)	163 (60.8%)	182 (60.7%)	133 (38.1%)
Once/week	374 (24.8%)	102 (29.1%)	83 (34.2%)	61 (22.8%)	51 (17.0%)	77 (22.1%)

Several times/week	298 (19.7%)	121 (34.6%)	38 (15.6%)	17 (6.3%)	31 (10.3%)	91 (26.1%)
Once/day	64 (4.2%)	36 (10.3%)	4 (1.6%)	2 (0.7%)	5 (1.7%)	17 (4.9%)
Several times/day	27 (1.8%)	22 (6.3%)	0 (0.0%)	0 (0.0%)	3 (1.0%)	2 (0.6%)

Note: Values are presented as n (% within cluster). Differences across clusters were tested using Pearson's chi-square test.
